# Effects of Prepolymerized Particle Size and Polymerization Kinetics on Volumetric Shrinkage of Dental Modeling Resins

**DOI:** 10.1155/2014/914739

**Published:** 2014-03-17

**Authors:** Tae-Yub Kwon, Jung-Yun Ha, Ju-Na Chun, Jun Sik Son, Kyo-Han Kim

**Affiliations:** ^1^Department of Dental Biomaterials, School of Dentistry, Kyungpook National University, 2-188-1 Samduk-dong, Jung-gu, Daegu 700-412, Republic of Korea; ^2^Department of Medical & Biological Engineering, Graduate School, Kyungpook National University, 2-188-1 Samduk-dong, Jung-gu, Daegu 700-412, Republic of Korea; ^3^Korea Textile Development Institute, 1083 Jungri-dong, Seo-gu, Daegu 703-712, Republic of Korea

## Abstract

Dental modeling resins have been developed for use in areas where highly precise resin structures are needed. The manufacturers claim that these polymethyl methacrylate/methyl methacrylate (PMMA/MMA) resins show little or no shrinkage after polymerization. This study examined the polymerization shrinkage of five dental modeling resins as well as one temporary PMMA/MMA resin (control). The morphology and the particle size of the prepolymerized PMMA powders were investigated by scanning electron microscopy and laser diffraction particle size analysis, respectively. Linear polymerization shrinkage strains of the resins were monitored for 20 minutes using a custom-made linometer, and the final values (at 20 minutes) were converted into volumetric shrinkages. The final volumetric shrinkage values for the modeling resins were statistically similar (*P* > 0.05) or significantly larger (*P* < 0.05) than that of the control resin and were related to the polymerization kinetics (*P* < 0.05) rather than the PMMA bead size (*P* = 0.335). Therefore, the optimal control of the polymerization kinetics seems to be more important for producing high-precision resin structures rather than the use of dental modeling resins.

## 1. Introduction

The acrylic family of polymers includes polymers and copolymers of acrylic and methacrylic acids and esters, acrylonitrile, and acrylamide [1]. However, most of the acrylic family products are acrylic and methacrylic esters. Acrylates are highly reactive due to the absence of the protecting methyl group at the vicinity of the double bond and may pose biocompatibility and shelf-life problems [2]. Moreover, polyacrylates are very soft because the polymer chains are not rigid [1]. Thus, methacrylate and its polymer, polymethacrylate, tend to be used in medical and dental applications to prepare shaped objects. Methyl methacrylate (MMA) is the most commonly used monomer in dentistry. Polymethyl methacrylate (PMMA) resin was originally introduced as a denture base material and was also formerly used as dental restorative materials [3]. They are now widely used for provisional crowns, fixed partial dentures, or orthodontic appliances and also for orthopedic surgery as bone cements [3].

Because of the very large (21 vol%) polymerization shrinkage, the polymerization of various PMMA products is carried out in stages to control the product dimensions for use in industrial applications [1, 4]. To prepare dental PMMA resins, a mixture of powdered polymer (prepolymerized PMMA particle) and monomer is used, and dissolution of the polymer in the monomer results in the formation of a plastic dough [5]. Along with this physical interaction, the resin is cured by the application of heat (heat-curing type) or chemicals (self-curing type). This mixed form enables ease of handling and minimizes shrinkage strain upon polymerization via the progressive substitution of the liquid monomer by the prepolymerized powder [3–5].

Dental modeling resins, which are also based on the PMMA/MMA system, have been developed for use in applications where highly precise resin structures are needed. As osseointegrated implants show little mobility relative to the surrounding bone, a misfit of implant-supported fixed partial dentures can allow the transmission of stress via the implants to the surrounding bone [6]. Therefore, dental laboratories use modeling or pattern resins for the construction of implant-retained suprastructures that require a precise fit. Although the manufacturers claim that these resins show little or no shrinkage, the actual data appears limited. Moreover, the mechanism of how the resins control or reduce polymerization shrinkage is unknown.

This study examined the effects of the prepolymerized PMMA particle size and polymerization kinetics on the volumetric shrinkage of five dental modeling resins and one temporary resin (control). The “linometer” method was used for determining the linear polymerization shrinkage, which was finally converted into a volumetric shrinkage. We hypothesized that (1) PMMA particles of the modeling resin are larger than that of the temporary resin and (2) the modeling resins yield lower final volumetric shrinkage values than the temporary resin.

## 2. Materials and Methods

### 2.1. Resin Materials Tested

In this study, Pi-Ku-Plast (PK; bredent GmbH & Co. KG, Germany), DuraLay (DL; Reliance Dental Mfg. Co., USA), Fino Resin PR (FR; Fino GmbH, Germany), GC Pattern Resin (GP; GC Corp., Japan), GC Pattern Resin LS (GL; GC America Inc., USA), and the control Jet Tooth Shade (JT; Lang Dental Mfg. Co. Inc., USA) were used. They all had similar chemical compositions: PMMA powder containing benzoyl peroxide (BPO) initiator, MMA liquid containing a cross-linking monomer, a tertiary amine coinitiator, and an inhibitor [3, 4].

### 2.2. Characterization of PMMA Particles

The morphology of the PMMA powders was observed by field-emission scanning electron microscopy (FE-SEM, JSM-6700F, Jeol, Japan) after platinum sputtering. The PMMA particle size was analyzed using a laser diffraction particle size analyzer (LA-950, Horiba, Japan) with a run length of 30 seconds [7]. Prior to the analysis, the powders were dispersed in ethanol and ultrasonicated for 3 minutes to ensure good particle dispersion [7].

### 2.3. Shrinkage Measurements

**Figure 1:**
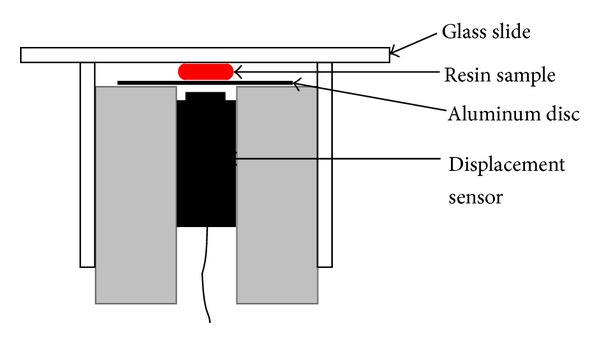
Schematic illustration of the linometer with a resin sample placed between the glass slide and aluminum disc.

Linear polymerization shrinkage measurements were performed using a custom-made linometer (R&B Inc., Korea) [8, 9]. A schematic illustration of the linometer is shown in Figure 1. All the resins were mixed at a powder/liquid (*P*/*L*) ratio of 3 : 1 by volume [4]. Freshly mixed materials were transferred to a Teflon mold to ensure that the same amount (~50 mm^3^) of resin was used for each linometer sample. Then, the materials were transferred to the aluminum disc in the linometer that had been previously coated with separating grease (Dow Corning, USA) then covered with a glass slide and loaded under constant pressure. The surface of the glass slide facing the specimen was also coated with the separating grease. As the resin under the slide glass was self-cured, the aluminum disc under the resin moved upward. The amount of disc displacement was measured using a sensor every 0.5 seconds for 20 minutes. The displacements were related to the true linear polymerization shrinkage because the surfaces to which the materials were attached were greased to allow a free shrinking movement in the radial sense along these surfaces [8]. Ten measurements were made for each resin.

The linear polymerization shrinkage was calculated using [8]:

(1) $$\begin{array}{c} \mathrm{lin}\%=\left[\frac{\Delta L}{L+\Delta L}\right]\times 100, \end{array}$$

where Δ*L* is the recorded displacement and *L* is the thickness of the specimen after polymerization. Finally, volumetric shrinkage was calculated using [8]:

(2) $$\begin{array}{c} \text{vol}\%=3\mathrm{lin}\%-0.03{\left(\mathrm{lin}\%\right)}^{2}+0.0001{\left(\mathrm{lin}\%\right)}^{3}. \end{array}$$

Two principal parameters were derived to express the polymerization shrinkage kinetics [10]: (1) the initial shrinkage, which is characterized as the percentage change in shrinkage in the first 10 seconds after a positive increase in shrinkage strain and (2) the overall time constant, the time for the shrinkage to achieve a fraction of 0.632 (or 1 − *e* ^−1^, which is derived from the Kohlrausch-Williams-Watts (KWW) stretched-exponential growth curve) of its final magnitude.

### 2.4. Statistical Analysis

For statistical analysis of the shrinkage data, one-way ANOVA and Tukey's post hoc test were used at *α* = 0.05. Polynomial regression was performed to determine the correlations between the final shrinkage strain and the two kinetics parameters (initial shrinkage and overall time constant) as well as the prepolymerized particle size.

## 3. Results and Discussion

The curing shrinkage of resin-based dental materials is measured using a variety of methods. These include dilatometric methods [11], the bonded disc method [4, 10, 12], the linometer method [8], and the strain-gauge method [4]. Although it is commonly used, dilatometry is very sensitive to the ambient temperature during the experiment because the volume of a medium in the dilatometer can increase as the temperature of the medium increases [12, 13]. On the other hand, the bonded disc method is relatively easy to use and does not require extensive and expensive instrumentation [11]. The linometer method is a modification of the bonded disc method and tracks the linear vertical displacement of a free floating aluminum disc fixed to the surface of a resinous material applied to a horizontal glass plate (Figure 1) [11, 13]. Like the bonded disc method, dimensional changes are confined to the thickness of the sample disc so that the fractional linear shrinkage approximates the volumetric shrinkage [8, 10]. Since the polymerization shrinkage strain can be reduced by the substitution of liquid monomer in a PMMA/MMA system by the prepolymerized powder [3–5], we hypothesize that the particle size is the main factor in decreasing the final volumetric shrinkage values in the dental modeling resins.

**Figure 2:**
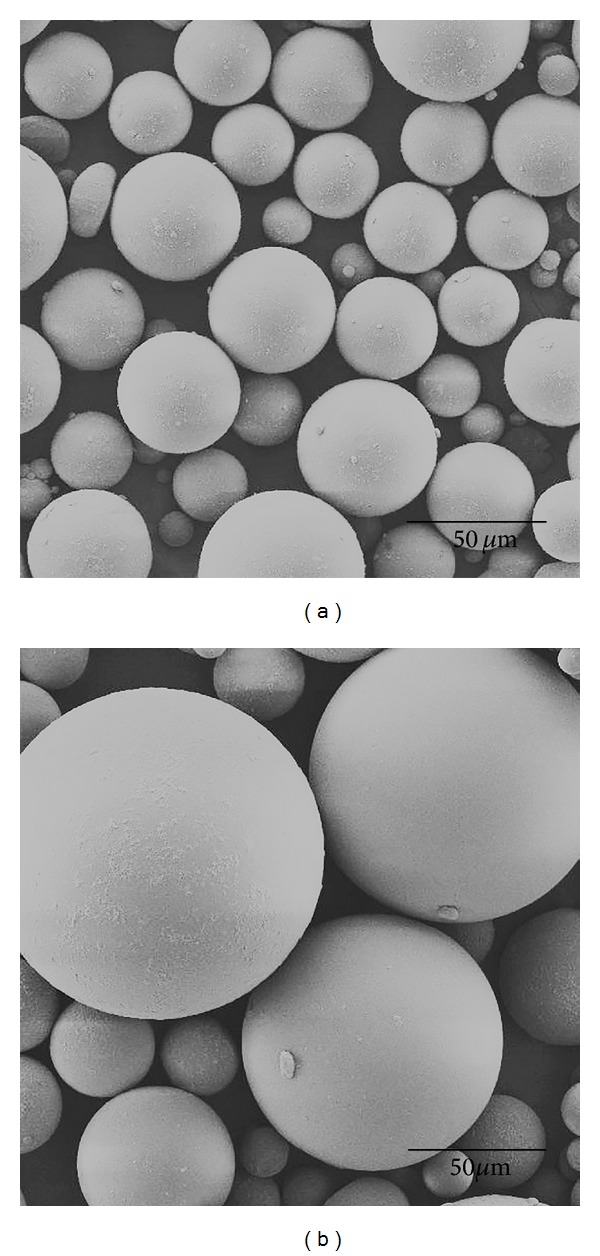
SEM images of the PMMA particles of the control (a) and GL (b) (original magnification 3000x). All particles tested in this study were spherical-shaped with different sizes.

**Figure 3:**
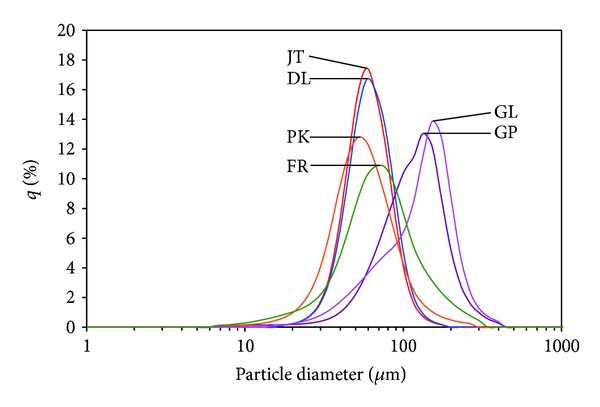
PMMA particle size distribution.

Figure 2 shows the representative SEM images of the PMMA particles of the temporary resin (control) and GL. In most dental acrylic resins, PMMA beads in the powder have diameters up to 100 *μ*m [14]. These are produced via suspension polymerization in which the MMA monomer, containing the initiator, is suspended as droplets in water [14]. In this study, all PMMA particles showed a spherical morphology (bead-shaped) with various sizes. The PMMA particle size distribution of each resin is shown in Figure 3.  Table 1 summarizes the median, mean, standard deviation, and mode of the particle sizes. Some differences between the median, mean, and mode indicate that the distributions are not completely symmetrical. Such trends were significant in FR, as indicated by the greater difference between the median and the mean than in the other resins. For GP and GL, large differences between the mode and the median or mean size indicate a bimodal distribution. The particle size of the modeling resins was similar to that of the control except for GP and GL, whose particle sizes were considerably larger. When powder and liquid are mixed, the MMA diffuses around and into the PMMA particles, releasing the prepolymerized polymer chains from the surface of the particles [3, 7]. A small particle size may improve the wetting of the beads and reduces the doughing time by forming a smoother mix and a greater dissolution of the particles [5]. However, this is only a physical interaction between the powder and liquid [3]. The tertiary amines (in the liquid) carry out the redox initiation together with BPO (in the powder) in a short period of time at room temperature [3, 15].

**Figure 4:**
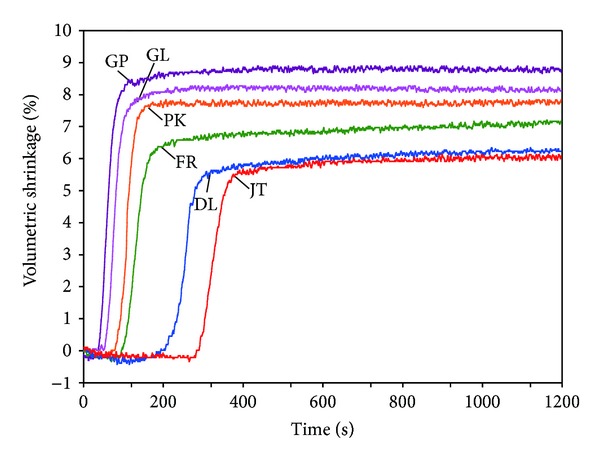
Representative volumetric shrinkage/time graphs (recorded 1 minute after starting the mix).

Figure 4 shows the representative polymerization shrinkage strain of the materials. Once initiated, the initial rigid polymerization shrinkage proceeded rapidly, as a nearly linear function of time [10]. Nonetheless, the normalized overall shrinkage response was approximately represented by the KWW stretched-exponential growth curve [10, 16]. This is particularly appropriate for the situation following the initial linear shrinkage [10]. Thus, the kinetic behavior was characterized by an overall time constant associated with the curve [10]. In addition, the initial shrinkage, indicating the initial reaction speed, was used to characterize the kinetic behavior [17].

Table 2 lists the statistical analysis results of the final volumetric shrinkage, initial shrinkage, and overall time constant. The mean final volumetric shrinkage-strain values ranged from 6.15% to 8.70%. The values for the modeling resins were similar (PK, DL, and FR; *P* > 0.05) or significantly greater (GP, GL; *P* < 0.05) than that of the control resin. This suggests that the modeling resins did not necessarily produce less polymerization shrinkage, but sometimes more shrinkage than the conventional PMMA/MMA resin when they were all mixed at the same *P*/*L* ratio. Both the initial shrinkage and the overall time constant also showed significant differences between the materials based on the one-way ANOVA (*P* < 0.001).

Volumetric shrinkage strain of a resin can be used to represent the extent of polymerization [18] because there is a direct relationship between the volumetric shrinkage and the monomer conversion [17]. In the PMMA/MMA resins tested in this study, the amounts of BPO initiator in the powder and amine coinitiator in the liquid are different between the materials. Thus, the different final shrinkage strains for the resins may have been mainly due to the different concentrations of the chemical initiation system (BPO/amine) in the resins [4, 18]. As shown in Figure 4, the onsets of polymerization shrinkage also differed significantly from each other, possibly because of the different types and concentrations of inhibitors present in the resins. During the induction or inhibition period, polymerization is halted by the chemical inhibitors. At the end of this period, when the inhibitor is consumed, polymerization proceeds at the same rate as in the absence of inhibitor [1]. However, higher inhibitor levels can compromise the final degree of conversion [19].

**Figure 5:**
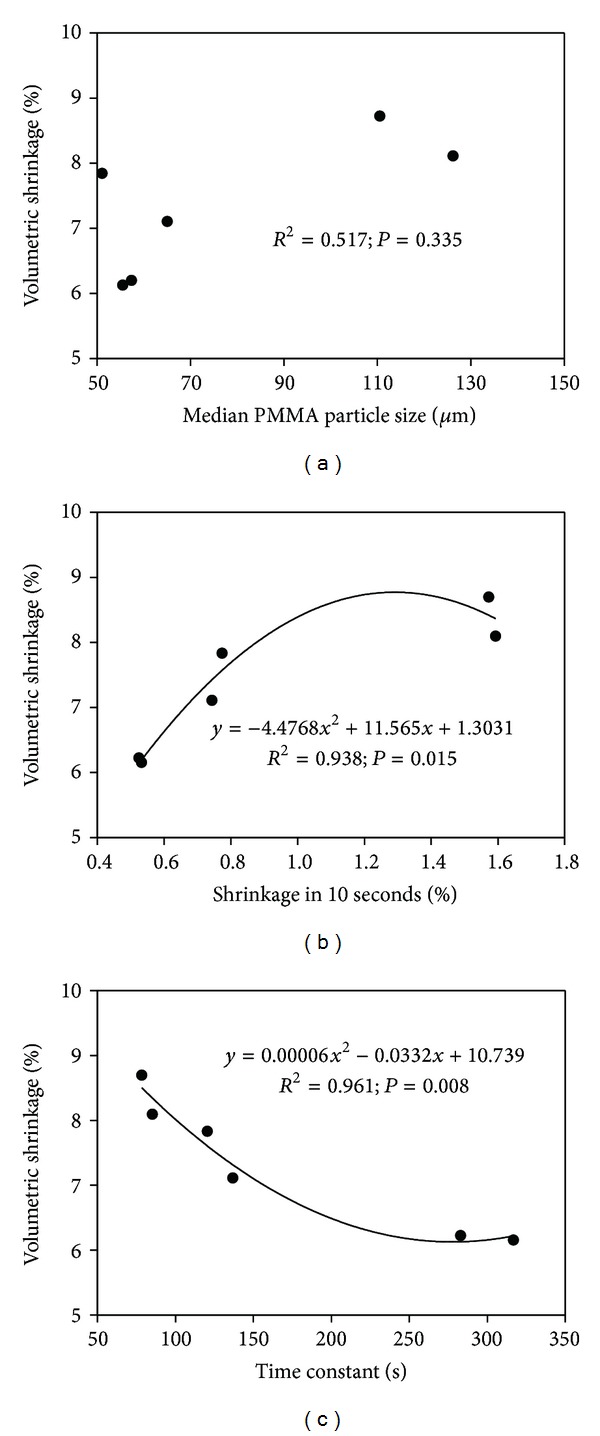
Polynomial regression curves of volumetric shrinkage versus the median particle size (a), initial shrinkage (b), and overall time constant (c).

Figure 5 shows the polynomial regression curves of volumetric shrinkage versus the median particle size, initial shrinkage, and overall time constant. In these statistical analyses, the median size was used because it is more commonly used and gives more meaningful information than the mean or mode size when using the laser diffraction technique. No statistical correlation was observed between the median PMMA particle size and the polymerization shrinkage in the present study (*P* = 0.335) (Figure 5(a)). Thus, although the use of prepolymerized powder in PMMA resins can reduce shrinkage strain upon polymerization [3–5], the particle size did not significantly influence the final shrinkage value. In contrast, the regression curves between the shrinkage and initial shrinkage (Figure 5(b)) and overall time constant (Figure 5(c)) fitted well with a second-order polynomial. In a study by Silikas et al. [4], when PMMA/MMA resin specimens were prepared with different *P*/*L* ratios by volume, the final shrinkage-strain values correlated positively with the *P*/*L* ratios. On the contrary, when an additional 1.0% BPO was added in the powder, the final shrinkage-strain values correlated negatively with the *P*/*L* ratio [4]. Based on these findings, the volumetric shrinkage strain seems to be more dependent on the extent of polymerization or degree of conversion rather than on the size or amount of PMMA beads in the PMMA/MMA mixture [18].

In general, polymerization shrinkage proceeds in two stages: pregelation and postgelation (or rigid) shrinkage [10, 20]. Shrinkage magnitudes obtained in this study are equal to or close to the postgelation volumetric shrinkage values [10, 13]. Some of the shrinkage occurs prior to the development of elastic properties in the resin, and some after the elastic behavior dominates [11]. During pregel polymerization, the resin may flow, allowing internal stresses to be relived [20]. After gelation, flow discontinues and cannot compensate for the polymerization shrinkage stresses [13]. Although postgel polymerization shrinkage strain and stress are more clinically relevant [20], the measurement of only postgel shrinkage strain could provide lower values than that of total shrinkage strain [13]. Although the dilatometry method is often believed to measure total contraction (pregel and postgel), adhesion of the specimen to a plate introduces constraint, which limits the collection of shrinkage [11]. A relatively new video-controlled technique may provide values close to the total shrinkage-strains values [13], and the data obtained in this study can be verified using such a technique.

The findings of this study require rejection of both the null hypotheses and suggest that dental modeling resins may not necessarily provide lower volumetric polymerization shrinkage than conventional PMMA/MMA resins. The manufacturers of modeling resins do not specify the *P*/*L* mixing ratios because the products are generally used in the brush-dip technique. When using the technique, the *P*/*L* ratio can be altered by the skillfulness of the dental laboratory technicians or dental clinicians. In such a case, changes in the amounts of the BPO initiator in the powder and amine coinitiator in the liquid can lead to changes in the rate of polymerization and, therefore, the final shrinkage-strain values [21]. In cases where resin samples are imperfectly cured, the measured shrinkage strain will be correspondingly reduced [10]. Also, the low degree of conversion can affect the mechanical properties of a resin material [19]. In the case of dental modeling resins, however, reducing the volumetric shrinkage by controlling the degree of conversion at the cost of the mechanical properties might be permissible to some extent. Nonetheless, possible adverse effects of changing *P*/*L* ratio, producing either excessive shrinkage strain or underpolymerization, should be understood and where possible controlled [4]. These findings may also be helpful in determining the optimal formulation of “true” low or nonshrinkage dental acrylic resins.

## 4. Conclusions

Within the limitations of this study, the following conclusions can be drawn.

1. The final volumetric shrinkage of dental PMMA/MMA resins, including modeling resins, was related to the polymerization kinetics rather than the prepolymerized PMMA particle sizes.
2. Dental modeling resins did not necessarily provide low volumetric shrinkage upon polymerization compared to the conventional PMMA/MMA resin when they were mixed at the same *P*/*L* ratio.

## Figures and Tables

**Table 1:** Volume-based PMMA particle size distribution.

Material	Median size*	Mean size^†^	SD^‡^	Mode size^§^
JT (Jet Tooth Shade, control)	55.41	58.28	19.51	55.06
PK (Pi-Ku-Plast)	51.00	57.41	30.35	48.32
DL (DuraLay)	57.27	60.17	20.37	55.42
FR (Fino Resin PR)	64.89	74.80	44.57	63.31
GP (GC Pattern Resin)	110.15	116.27	53.74	124.80
GL (GC Pattern Resin LS)	125.71	127.12	59.70	143.32

All values are in *μ*m. *The size that splits the volume distribution with half above and half below this diameter; ^†^the volume mean diameter; ^‡^standard deviation for the frequency distribution;  ^§^the peak of the frequency distribution.

**Table 2:** Shrinkage data determined using a linometer.

Material	Volumetric shrinkage (%)	Shrinkage in 10 seconds (%)	Overall time constant (s)
JT	6.15 ± 0.64 A	0.53 ± 0.09 A	316.82 ± 23.36 A
PK	7.83 ± 1.57 AB	0.77 ± 0.18 A	120.56 ± 19.39 B
DL	6.22 ± 1.24 A	0.52 ± 0.10 A	283.01 ± 28.91 C
FR	7.11 ± 0.86 AB	0.74 ± 0.12 A	136.79 ± 19.41 B
GP	8.70 ± 1.78 B	1.57 ± 0.27 B	78.43 ± 14.03 D
GL	8.09 ± 1.91 B	1.59 ± 0.29 B	85.27 ± 6.85 D

Values are expressed as mean ± standard deviation. Within a row, values followed by different uppercase letters are statistically different (*P* < 0.05).
